# Whole Grain Intakes in the Diets Of Malaysian Children and Adolescents – Findings from the MyBreakfast Study

**DOI:** 10.1371/journal.pone.0138247

**Published:** 2015-10-16

**Authors:** Norimah AK, H. C. Koo, Hamid Jan JM, Mohd Nasir MT, S. Y. Tan, Mahendran Appukutty, Nurliyana AR, Frank Thielecke, Sinead Hopkins, M. K. Ong, C. Ning, E. S. Tee

**Affiliations:** 1 School of Healthcare Sciences, Faculty of Health Sciences, Universiti Kebangsaan Malaysia, Kuala Lumpur, Malaysia; 2 Department of Health Professional and Food Service, Faculty of Health and Life Sciences, Management & Science University, Shah Alam, Malaysia; 3 Nutrition Programme, School of Health Sciences, Universiti Sains Malaysia, Kubang Kerian, Kelantan, Malaysia; 4 Department of Nutrition and Dietetics, Faculty of Medicine and Health Sciences, Serdang, Universiti Putra Malaysia, Kembangan, Malaysia; 5 Department of Nutrition and Dietetics, School of Health Sciences, International Medical University, Kuala Lumpur, Malaysia; 6 Sports Science Programme, Faculty of Sports Science and Recreation, Universiti Teknologi MARA, Shah Alam, Malaysia; 7 Cereal Partners Worldwide, Lausanne, Switzerland; 8 Nestlé Research Center, Vers chez les Blanc, Lausanne, Switzerland; 9 Nestle R&D Center, Singapore, Singapore; 10 Nutrition Society of Malaysia, c/o Division of Human Nutrition, Institute for Medical Research, Kuala Lumpur, Malaysia; TNO, NETHERLANDS

## Abstract

**Background:**

Diets rich in whole grain are associated with several health benefits. Little is known however, about whole grain consumption patterns in Malaysia. The aim of this study was to assess whole grain intakes and dietary source in Malaysian children and adolescents.

**Methods:**

This analysis is from the MyBreakfast study, a national cross sectional study investigating eating habits among primary and secondary school children throughout Malaysia, conducted in 2013. Children (n = 5,165) and adolescents (n = 2,947) who completed two days of dietary assessment using a food record or recall respectively were included. The whole grain content of foods was estimated mainly through the use of quantitative ingredient declarations on food labels. All wholegrain foods were considered irrespective of the amount of whole grain they contained.

**Results:**

Overall, only 25% of children and 19% of adolescents were wholegrain consumers. Mean daily intakes in the total sample were 2.3g/d (SD 5.8g/d) in children and 1.7g/d (SD 4.7g/d) in adolescents and in the consumer’s only sample, mean intakes reached 9.1g/d (SD 8.6) and 9.2g/d (SD 7.1g/d) respectively. Wheat was the main grain source of whole grain while ready to eat breakfast cereals and hot cereals were the main food contributors. Less than 3% of the children and adolescents reached the US quantitative whole grain recommendation of 48g/day.

**Conclusion:**

Whole grain is consumed by only a minority of Malaysian children and adolescents and even among consumers, intakes are well below recommendations. Efforts are needed to firstly understand the barriers to whole grain consumption among Malaysian children in order to design effective health promotion initiatives to promote an increase in whole grain consumption.

## Introduction

A growing body of epidemiological evidence indicates that consumption of whole grain may be protective against several chronic diseases in adults including diabetes [1–2], obesity [3–4], cardiovascular disease [2, 5–6] and colorectal cancer [7]. In children and adolescents, the association between consumption of whole grain and health benefits has been less explored but several studies have reported improved nutrient intakes and diet quality among whole grain consumers compared to non-consumers [8–11]. Furthermore, consumption of more than 1 serving/day (>16g/d) of whole grain compared to less than 1 serving/day was associated with a significantly lower waist circumference and body mass index (BMI) in a group of American adolescents [12], while more than 1.5 servings/day (>24g/d) was associated with a lower BMI z score and risk of obesity in a rural sample of American children [13].

Although there is no globally accepted definition of whole grain, The American Association of Cereal Chemists International (AACCI) definition has been widely adopted. The AACCI defines a whole grain as consisting of the intact, ground, cracked or flaked caryopsis, whose principal anatomical components, the starchy endosperm, germ and bran are present in the same relative proportions as they exist in the intact grain [14]. Recently, the consortium of the HEALTHGRAIN EU project extended this definition by allowing for small losses to the grain (<2%) and bran (<10%) to reflect current milling practices [15]. In addition to being a source of carbohydrates (concentrated mainly in the endosperm), whole grains are a rich source of dietary fiber, vitamins, minerals, phytoestrogens and antioxidants (concentrated mainly in the bran and germ) [16].

A recommendation to eat whole grain is included in the dietary guidelines of many countries for both children and adults; however, the exact recommendation varies widely between countries. For example, quantitative recommendations exist in the US (≥48g/d) [17] and Scandinavia (≥75g/10MJ) [18], while Singapore provides a semi quantitative advice of “Include at least one daily serving of wholegrain products such as brown rice or wholemeal bread” [19], and a much more generic guidance is given in the United Kingdom—“choose whole grain varieties when you can” [20]. The Malaysian Dietary Guidelines for Children and Adolescents recommends four to nine servings of cereal foods per day according to age and that at least half of the grain servings should be from whole grain sources [21].

Despite these recommendations, data on whole grain intakes at a population level are only available for a handful of countries worldwide. Most of the existing data comes from European and US-based studies, which indicate a variable whole grain intake among children and adolescents ranging from only 4–5g/d in France [10] to 54g/d in Denmark [22]. To our knowledge, the only published data on whole grain consumption in the Southeast Asia region relates to Singaporean adults, in which the average intake was 26g per day in 2010 [23]. Therefore, the aim of the present study was to determine the intake of whole grain in the diets of Malaysian children and adolescents, to identify the key sources of whole grain and to assess the extent to which compliance with a quantitative whole grain recommendation has been met.

## Methodology

### Study design and respondents

The present analysis is from the MyBreakfast study, a national cross-sectional study investigating eating habits among Malaysia primary and secondary school children, conducted from April to October 2013 by the Nutrition Society of Malaysia. Permission to carry out data collection was granted by the Ministry of Education Malaysia and the Department of Education in each of the states involved. The study protocol was reviewed and approved by the Universiti Kebangsaan Malaysia research ethics committee and was conducted in accordance with the Declaration of Helsinki. A study information sheet was sent to parents together with a standard consent form from UKMREC. A verbal explanation on the study was also given to the participatns at school. For children aged 6 to 9 years, a written informed consent was obtained from parents, while for children aged 10 to 17 years, written informed consents were obtained from both parents and participants prior to the study.

**Fig 1:**
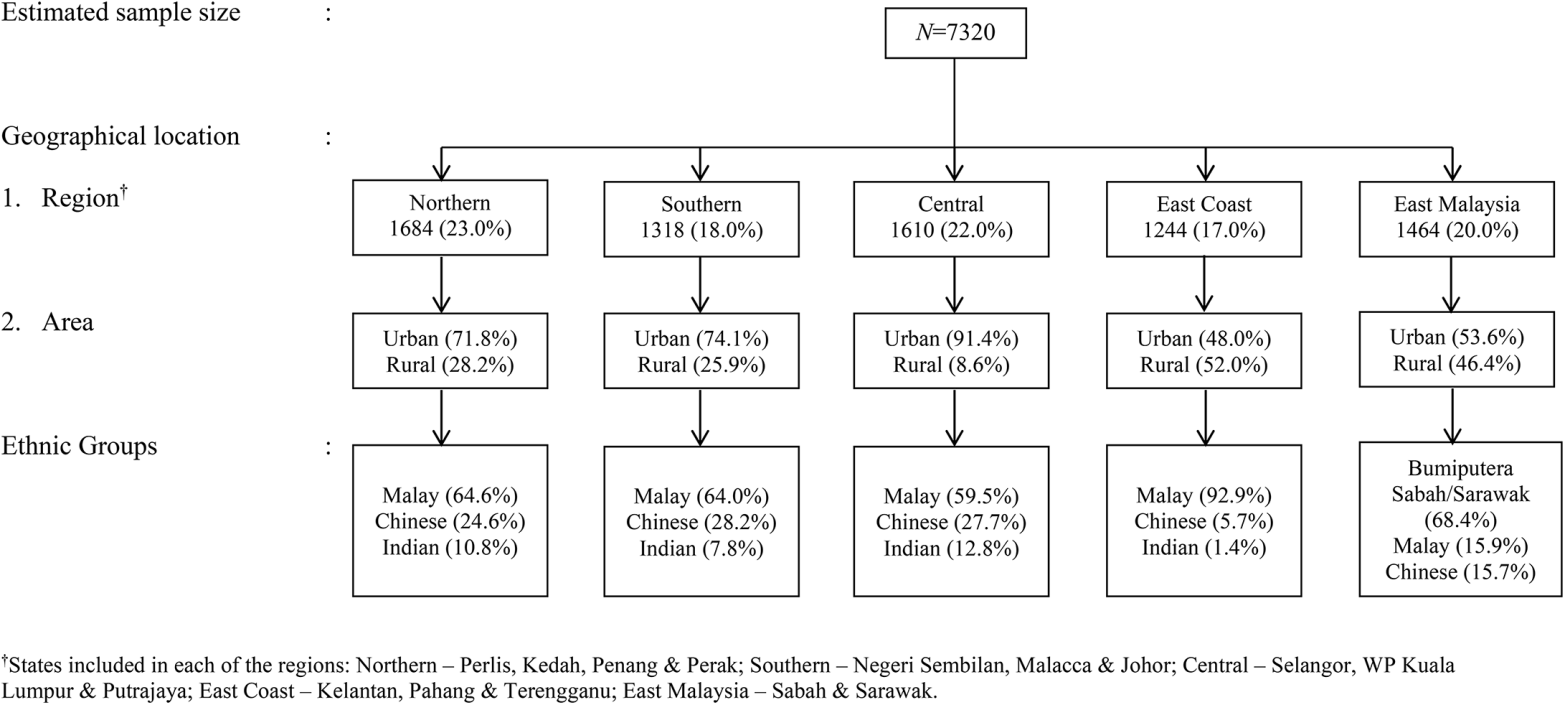
Sampling procedure. ^†^States included in each of the regions: Northern–Perlis, Kedah, Penang & Perak; Southern–Negeri Sembilan, Malacca & Johor; Central–Selangor, WP Kuala Lumpur & Putrajaya; East Coast–Kelantan, Pahang & Terengganu; East Malaysia–Sabah & Sarawak.

A multistage sampling method of respondents aged 6 to 17 years was carried out, based on geographical location and ethnic group distribution. The estimated sample size was calculated based on the total population of children aged 6 to 12 years (n = 3,414,906) and 13–17 years (n = 2,521,688) in Malaysia derived from the Population and Housing Census 2010 [24]. From this census data, the percentage of children required in each of the five regions, namely Northern, Southern, East Coast, Central region and East Malaysia were determined and the proportion of children in the urban and rural areas of each state of the regions and by ethnic group was calculated as outlined in Fig 1. A standardized ratio of 1:1 for gender was used to determine the number of girls and boys required for each ethnicity in the rural and urban area. Equal numbers of children and adolescent aged 6 to 17 years were invited to participate in this study. Inclusion criteria were apparently healthy Malaysian school children and adolescents aged 6 to 17 years old, without physical or mental disabilities and present in school on the day of assessment with parental consent.

The lists of public primary and secondary schools registered in each of the states as of 31^st^ of January 2011 were obtained from the Ministry of Education. Twenty-eight primary schools and 56 secondary schools were randomly selected from rural and urban area, respectively. For each school, two to three classes of Primary 1 to Primary 5, and Secondary 1, 2 and 4 were randomly recruited. Students from Primary 6, Secondary 3 and Secondary 5 were not selected for participation as permission was not granted for these groups who were candidates enrolled in the 3 national level examinations. A total of 13,694 children and adolescents were invited to participate in the study and 9,369 agreed to take part, resulting in a response rate of 68.4%. Of the 9,369 participants who agreed to take part, 664 had no dietary data and a further 593 were missing 2 complete days of dietary recall or record, resulting in a final sample size of 8,112. The percentage of missing and incomplete dietary data was 10.7% among the primary school children and 15.5% among the secondary school children.

### Data collection procedure

Firstly, a study information sheet, consent form and a questionnaire on socio-demographic background was sent to the parents. The socio-demographic questionnaire included questions on the respondent’s date of birth, sex, ethnicity, father’s education level and monthly household income. One week was given to the parents to return the consent form and the socio-demographic questionnaire if they agreed to participate in the study.

### Dietary intake assessment

Daily food and beverage intakes were assessed on one weekday and one weekend day using a food record for respondents aged 6 to 9 years and a 24-hour dietary recalls for respondents aged 10 to 17 years. Parents completed the food record for respondents aged 6 to 9 years with the assistance of an instruction sheet. A brief instruction was also sent to the parents through short messaging system (SMS). Parents were requested to record all foods and beverages consumed by their child over the day and to note the time of consumption. The instruction sheet outlined common household measures to assist parents’ in the estimation of portion size consumed. Upon return of the dietary records in school, any incomplete questionnaires were sent back to their parents with a short note on the uncompleted part along with a SMS. Meanwhile, a two-day 24-hour dietary recall was conducted for children aged 10 to 17 years in a one-to-one interview by trained researchers in school. Children were requested to recall all the foods and beverages consumed in the past 24-hour. Household items such as bowls, dishes, spoons and glasses in commonly-used sizes, food models and pictures of common foods were used to facilitate portion sizes estimation. For the dietary record and recall, brand information and cooking method were also recorded where applicable.

### Calculation of whole grain intakes

The AACCI definition of whole grain as outlined in the introduction was applied to the current study. Wholegrain foods were defined as foods made with at least one whole grain ingredient from the following grains: wheat, rice, oats, maize, rye and barley. There was no lower limit applied to the whole grain content of a food for inclusion in the analysis. From 1,931 unique foods reported to be consumed in the survey, only 49 of them were identified as foods made with whole grain ingredients, of which 47 were recorded at brand level. The wholegrain foods identified were grouped into one of the seven groups: Ready-to-eat cereals (RTEBC) (n = 16), Bread (n = 5), Hot cereal (n = 6), Pasta/noodle (n = 4), Rice (n = 7), Biscuits (n = 4) and Others (n = 7). The food group “Others” consisted of cereal bars, chapatti and mixed dishes containing whole grain. The amount of whole grain per 100g in each wholegrain food was estimated by one of three methods 1) using quantitative ingredient declarations on food package labels (n = 38) 2) directly contacting the manufacturers to obtain this information (n = 10) or 3) taking an average of the whole grain content of similar products (n = 1). Details of all wholegrain foods including the total whole grain content per 100g, whole grain content per 100g by grain type and the method of calculation were recorded in an excel spreadsheet. For each participant, the amount of whole grain consumed was estimated by multiplying the actual weight of each whole grain food consumed by the estimated percentage whole grain content. Mean daily intakes over the two days of recall or record were calculated. Whole grain consumers were defined as respondents who consumed a wholegrain food on at least one of the recall or record days. As stated previously, the Malaysian dietary guidelines for children and adolescents do not include quantitative recommendations for whole grain intake but advise that “half of grain servings should come from whole grain” [21]. As the minimum recommended number of grain servings is 4 per day, the whole grain servings should be at least 2 per day. By using the recommended serving size for common grain sources among Malaysians including rice, bread, biscuits and RTEBC [25] and applying an average whole grain content for each food based on the whole grain database created in the current study we estimated that the Malaysian recommendation for whole grain equates to a minimum of 43 g per day. As this is a crude estimation and similar to the US recommendations; we chose to compare whole grain intakes to various levels of the US quantitative recommendation of 48 g/day [17].

### Statistical analyses

Statistical analyses were conducted using the SPSS version 22.0 (IBM SPSS Statistics, 2014). Data were entered, cleaned and checked before data analyses. Categorical data were presented as number and percentage. Continuous data were presented as mean and standard deviation or median and percentiles. Univariate analyses were carried out using descriptive statistic. The association between categorical variables and whole grain consumer groups were determined using chi-square test. The differences between the whole grain intakes across socio-demographic variables were determined using Kruskal-Wallis test or Mann-Whitney U test, as the data were not normally distributed. Two sided *p<*0.05 was considered statistically significant at 95% confidence interval.

## Results

### Whole grain intakes

Tables 1 and 2 present daily whole grain intakes (g/d) among Malaysian children and adolescents by socio-demographics for the total sample and consumers only. A total of 5,165 children and 2,947 adolescents participated in the study, with a slightly higher proportion of girls (55.1% in children and 51.8% in adolescents) than boys. A majority of the respondents were Malay (65.0% in children and 55.9% in adolescents), came from the Northern region (26.4% in children and 22.9% in adolescents) and lived in urban areas (67.1% in children and 69.3% in adolescents) which is generally reflective of the 2010 Population and Housing Census of Malaysia [24]. Overall, only 24.9% of children and 18.8% of adolescents were wholegrain consumers. Mean daily intakes in the total sample were 2.3g/d and 1.7g/d for children and adolescents, respectively. In consumers only, mean daily intakes were 9.1g/d for children and 9.2g/d for adolescents, while median intakes were 7.0g/d and 8.1g/d in children and adolescents, respectively. Among children, the proportion of whole grain consumers tended to be highest in younger children aged 6–9 years, those from the Central, in urban areas, those of Indian ethnicity and among those from families with a monthly income > RM3500, as well as a higher level of education (*p* = 0.001). Among adolescents, there were more female whole grain consumers than males (*p* = 0.036) and more Bumiputera Sabah/ Sarawak consumers than other ethnicities (*p* = 0.012). Similar to children, there were more adolescent whole grain consumers in the Central and East Malaysia region, in urban areas and from higher income families, as well as a higher level of education (*p* = 0.001).

**Table 1:** Whole grain intakes (g/day) among Malaysian children by socio-demographics.

	Total Sample				Whole grain consumers					
	n (%)	Mean±SD	P95	*p*-value^f^	n (%)	Mean±SD	Median	P5-P95	*p*-value^e^	*p*-value^f^
**Overall**	5165 (100.0)	2.3±5.8	12.3		1286 (24.9)	9.1±8.6	7.0	2.2–25.9		
**Age**				0.001					0.001	0.001
6–9 years	2873 (55.6)	2.2±5.1	12.2		784 (27.3)	8.2±6.7	5.4	2.2–21.7		
10–12 years	2292 (44.4)	2.3±6.6	13.4		502 (21.9)	10.4±10.7	8.1	2.7–30.0		
**Sex**				NS					NS	NS
Boys	2318 (44.9)	2.4±6.3	12.5		579 (25.0)	9.7±9.5	8.1	2.9–28.0		
Girls	2847 (55.1)	2.1±5.4	12.1		707 (24.8)	8.6±7.8	6.1	2.0–24.7		
**Regions**				0.001					0.001	0.001
Central	976 (18.9)	3.3±7.1^a^	15.0		320 (32.8)	9.9±9.4^a^	8.1	2.2–27.0		
Southern	1003 (19.4)	2.6±6.2^b^	13.4		277 (27.6)	9.4±8.6^a^ ^,^ ^b^	8.1	4.0–26.4		
Northern	1365 (26.4)	1.7±5.6^c^	8.9		262 (19.2)	8.9±9.8^b^ ^,d^	6.3	2.7–24.5		
East Coast	927 (17.9)	1.7±4.7^c^	8.3		190 (20.5)	8.1±7.3^c^ ^,d^	4.1	2.0–27.1		
East Malaysia	894 (17.3)	2.2±5.0^b^	11.5		237 (26.5)	8.3±6.5^d^	5.4	2.0–20.8		
**Area**				0.001					0.001	NS
Urban	3468 (67.1)	2.5±6.2	13.5		938 (38.0)	9.3±8.9	7.6	2.7–25.9		
Rural	1697 (32.9)	1.7±4.8	10.5		348 (20.5)	8.4±7.4	5.4	2.0–25.0		
**Ethnic groups**				0.001					0.001	NS
Malay	3359 (65.0)	2.1±5.4^a^	12.2		807 (24.0)	8.7±8.0	6.1	2.2–25.6		
Chinese	814 (15.8)	2.3±7.0^a^	13.5		173 (21.3)	11.0±12.1	8.1	2.3–33.6		
Indian	373 (7.2)	3.0±5.8^b^	14.1		130 (34.9)	8.7±6.9	7.5	4.1–24.5		
Bumiputera Sabah/Sarawak	573 (11.1)	2.4±5.7^a^	12.2		156 (27.2)	8.9±8.2	7.4	2.2–21.3		
Others	46 (0.9)	4.9±8.4^c^	25.1		20 (43.5)	11.3±9.6	8.1	2.1–39.9		
**Monthly income (RM)**				0.001					0.001	NS
<1500	1656 (33.0)	1.9±5.1^a^	10.8		351 (21.2)	8.8±7.7	6.1	2.2–25.8		
1501–3500	1442 (28.7)	2.2±5.4^b^	12.2		349 (24.2)	9.0±7.8	6.6	2.7–27.0		
3501–5500	913 (18.2)	2.4±6.5^b^	12.3		239 (26.2)	9.1±10.1	8.1	2.0–22.4		
5501–7500	497 (9.9)	2.3±5.1^b^	13.5		135 (27.2)	8.5±6.6	5.4	2.7–24.3		
>7500	517 (10.3)	3.2±7.8^c^	16.2		165 (31.9)	10.1±11.0	8.1	2.9–28.5		
**Father’s Education Level**										
Primary education*	339 (7.0)	1.5±4.9^a^	10.8	0.001	56 (16.5)	9.4±7.0	8.1	3.5–27.1	0.001	NS
Secondary education	2781 (56.9)	2.0±5.0^b^	10.8		638 (22.9)	8.5±7.3	5.4	2.2–25.9		
Tertiary education	1765 (36.1)	2.8±6.9^c^	13.5		518 (29.3)	9.6±9.9	8.1	2.2–25.5		

NS: no significant difference; RM: Ringgit Malaysia

^a,b,c^ different alphabets denote significant difference (Kruskal Wallis test or Mann-Whitney U test)

^e^ comparison of whole grain consumer distribution by socio-demographic background (chi-square)

^f^ comparison of whole grain mean intake by socio-demographic background (Kruskal Wallis test or Mann-Whitney U test)

*Respondents’ fathers from total sample (n = 48) and whole grain consumers (n = 7) had no formal education, and due to the small sample size for analyses, they are included in the primary education group

For total sample, 140 missing data for monthly income variable; 280 missing data for father’s education level variable

For whole grain consumers, 182 missing data for monthly income variable; 74 missing data for father’s education level variable

**Table 2:** Whole grain intakes (g/day) among Malaysian adolescents by socio-demographics.

	Total sample				Whole grain consumers					
	n (%)	Mean±SD	P95	*p*-value^e^	n (%)	Mean±SD	Median	P5-P95	*p*-value^d^	*p*-value^e^
**Overall**	2947 (100)	1.7±4.7	10.8		555 (18.8)	9.2±7.1	8.1	3.3–23.1		
**Age**				NS					NS	NS
13–15 years	2326 (78.9)	1.7±4.8	10.8		432 (18.6)	9.3±7.4	8.1	3.3–24.3		
16–17 years	621 (21.1)	1.7±4.4	10.8		123 (19.8)	8.6±6.1	8.1	2.9–21.1		
**Sex**				0.047					0.036	NS
Boys	1419 (48.2)	1.6±4.6	10.8		245 (17.3)	9.4±7.3	8.1	2.9–25.3		
Girls	1528 (51.8)	1.8±4.8	10.8		310 (20.3)	9.0±7.1	8.1	2.9–21.5		
**Regions**				0.001					0.001	NS
Central	635 (21.5)	2.4±4.7^a^	10.8		186 (29.3)	7.7±4.9	8.1	1.2–18.7		
Southern	580 (19.7)	1.7±5.4^b^	11.5		84 (14.5)	11.6±9.7	8.1	3.5–25.7		
Northern	676 (22.9)	1.4±4.1^b^	10.5		95 (14.1)	9.7±6.2	8.1	2.8–22.1		
East Coast	467 (15.8)	1.1±4.5^b^	8.1		53 (11.3)	9.8±9.9	4.6	1.8–32.6		
East Malaysia	589 (20.0)	2.0±4.7^c^	10.8		137 (23.3)	8.7±6.6	7.9	3.5–24.9		
**Area**				0.001					0.001	NS
Urban	2041 (69.3)	1.9±5.1	10.8		433 (21.2)	9.4±7.5	8.1	3.3–24.7		
Rural	906 (30.7)	1.1±3.4	8.1		122 (13.5)	8.3±5.6	7.4	2.7–21.2		
**Ethnic groups**				0.008					0.012	NS
Malay	1647 (55.9)	1.4±3.8^a^	10.4		280 (17.0)	8.4±5.3	8.1	3.4–19.3		
Chinese	639 (21.7)	1.8±5.3^a^	10.7		123 (19.2)	9.9±8.5	8.1	1.7–27.0		
Indian	277 (9.4)	2.4±6.7^b^	3.9		58 (20.9)	11.2±10.7	8.1	2.5–39.7		
Bumiputera Sabah/Sarawak	373 (12.7)	2.3±5.5^b^	10.8		92 (24.7)	9.4±7.4	8.1	3.6–28.3		
Others	11 (0.4)	1.5±3.3^a^	-		2 (18.2)	8.2±0.1	8.2	-		
**Monthly income (RM)**				0.001					0.001	NS
<1500	1096 (39.8)	1.2±3.8^a^	10.2		159 (14.5)	8.6±6.2	8.0	3.5–19.2		
1501–3500	787 (28.6)	1.6±4.9^a^	10.8		128 (16.3)	8.6±7.9	8.1	2.8–24.8		
3501–5500	413 (15.0)	2.3±6.0^b^	12.8		92 (22.3)	10.2±9.1	8.1	2.8–31.7		
5501–7500	211 (7.7)	2.6±5.3^b^	14.4		61 (28.9)	8.9±6.5	8.1	1.0–25.4		
>7500	248 (9.0)	1.9±4.0^b^	11.4		61 (24.6)	7.6±4.5	7.4	1.1–16.8		
**Father’s Education Level**				0.001					0.001	NS
Primary education*	233 (8.8)	1.6±4.7^a^ ^,^ ^b^	10.5		42 (18.0)	9.1±7.3	8.1	3.5–26.0		
Secondary education	1594 (60.1)	1.5±4.6^a^ ^,^ ^b^	10.5		255 (16.0)	9.4±7.4	8.1	3.6–22.1		
Tertiary education	827 (31.1)	2.2±5.1^a^ ^,^ ^c^	12.2		200 (24.2)	8.9±6.9	8.1	1.8–25.5		

NS: no significant difference; RM: Ringgit Malaysia

^a,b,c^ different alphabets denote significant difference (Kruskal Wallis test or Mann-Whitney U test)

^d^ comparison of whole grain consumer distribution by socio-demographic background (chi-square)

^e^ comparison of whole grain mean intake by socio-demographic background (Kruskal Wallis test or Mann-Whitney U test)

* Respondents’ fathers from total sample (n = 28) and whole grain consumers (n = 8) had no formal education, and due to the small sample size for analyses, they are included in the primary education group

For total sample: 192 missing data for monthly income variable; 293 missing data for father’s education level variable

For whole grain consumers: 54 missing data for monthly income variable; 58 missing data for father’s education level variable

Mean intakes of whole grain tended to increase with age, although this was only significant in children, with values increasing from 2.2g/d (6–9 years) to 2.3g/d (10–12 years) in the total population (*p* = 0.001), and from 8.2g/d (6–9 years) to 10.4g/d (10–12 years) in consumer only (*p* = 0.001). In both children and adolescents, whole grain intakes in the total sample were significantly higher in the Central region, urban areas and among those from families with a higher income and education level (*p* = 0.001). In the total children’s sample, intakes tended to be higher among Indians (*p* = 0.001) and for the adolescents, intakes were higher among Indians and Bumputera Sabah/ Sarawak (*p* = 0.008). These significant differences in whole grain intakes by demographics tended to disappear when the consumer only sample was examined.

### Sources of whole grain intake

**Fig 2:**
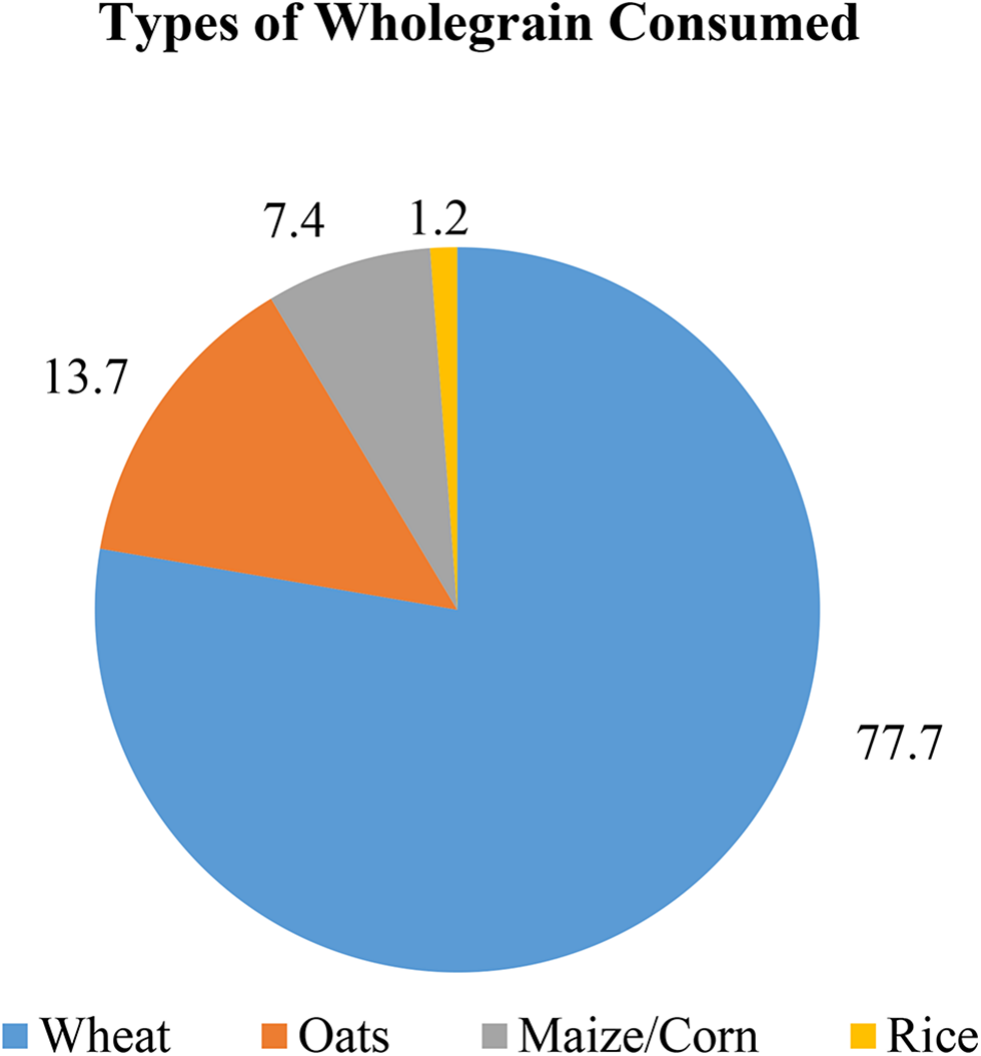
Distribution of types of whole grains consumed by a sample of 1,286 Malaysian children aged 6–12 years and 555 adolescents aged 13–17 years who were whole grain consumers. The main contributor was wheat (77.7%), followed by oats (13.7%), maize/ corn (7.4%) and rice (1.2%).

**Fig 3:**
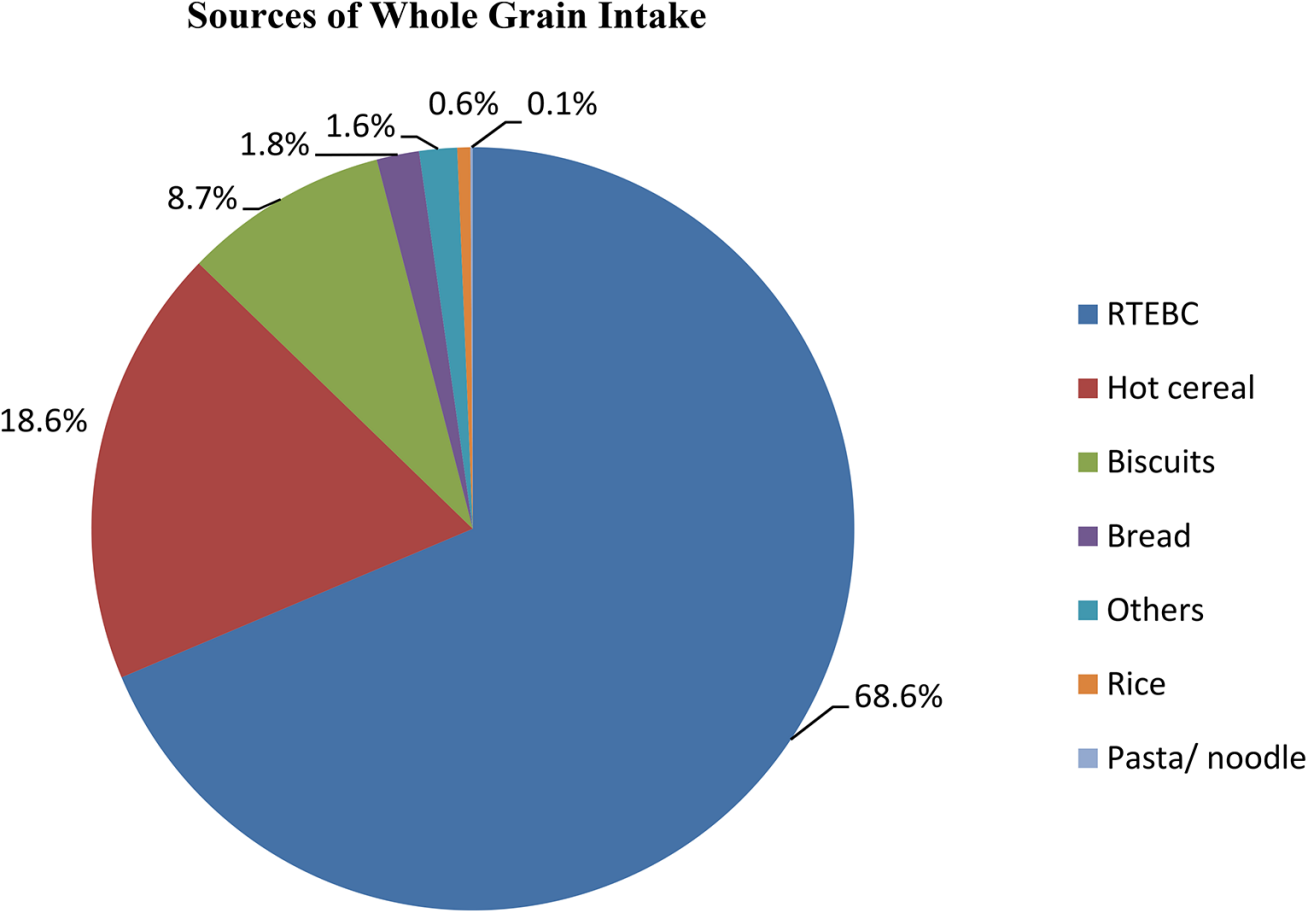
Distribution of sources of whole grain intake among 1,286 Malaysian children aged 6–12 years who were whole grain consumers. The major food source of whole grain intake was ready-to-eat breakfast cereal (68.6%), followed by hot cereal (18.6%), biscuits (8.7%), bread (1.8%), others (1.6%), rice (0.6%) and pasta/noodle (0.1%).

**Fig 4:**
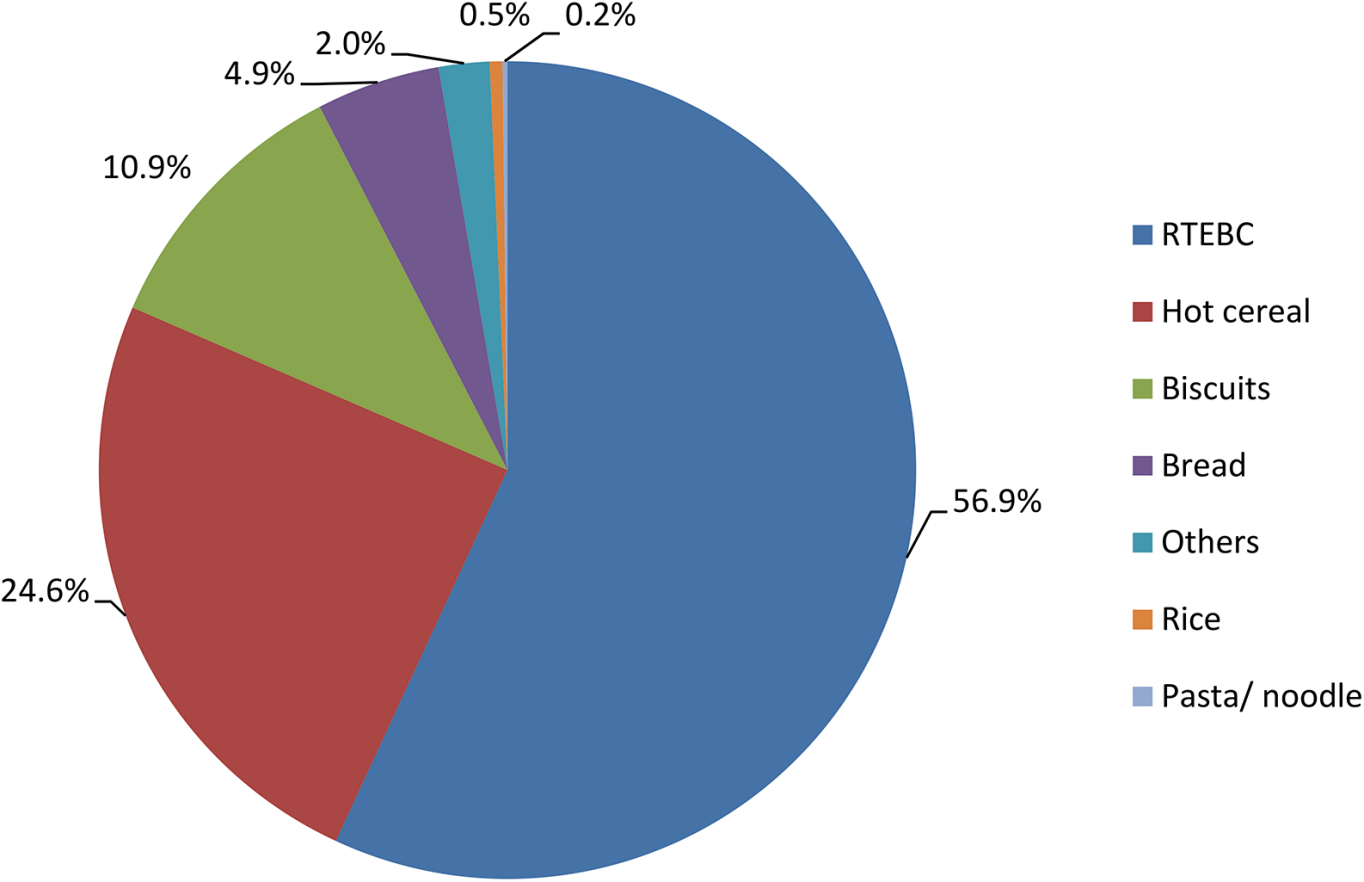
Distribution of sources of whole grain intake among 555 adolescents aged 13–1 years who were whole grain consumers. The major food source of whole grain intake was ready-to-eat breakfast cereal (56.9%), followed by hot cereal (24.6%), biscuits (10.9%), bread (4.9%), others (2.0%), rice (0.5%) and pasta/noodle (0.2%).

Wheat was the major grain contributing to whole grain intake, providing 77.7% of the total daily whole grain intake in consumers. Oat was the second highest contributor (13.7%), followed by maize/corn (7.4%) and a minimal contribution from rice (1.2%) (Fig 2). The percentage contribution of the seven food groups to the total whole grain intake for children and adolescents who consumed whole grain are presented in Figs 3 and 4. For both children and adolescents, the major food source of whole grain intake was RTEBC (68.6% and 56.9%, respectively), followed by hot cereal (18.6% and 24.6%, respectively), biscuits (8.7% and 10.9%, respectively), bread (1.8% and 4.9%, respectively), other (1.6% and 2.0%, respectively), rice (0.6% and 0.5%, respectively) and pasta/ noodle (0.1% and 0.2%, respectively). Overall, RTEBC made a greater contribution to whole grain intakes in boys (67.4%) compared to girls (62.2%) while girls (22.0%) had a greater contribution from hot cereals than boys (18.5%). RTEBC was the major source of whole grain for all regions followed by hot cereals and biscuits with very little difference between urban and rural areas. RTEBC contributed more than 60% to whole grain intakes in Malays, Indians and Bumiputera Sabah/ Sarawak compared to only 43.4% in the Chinese. Hot cereals on the other hand contributed 36.3% to whole grain intakes in the Chinese and <22% in the other ethnic groups.

### Whole grain intake and dietary recommendations

The comparison of whole grain intake in children and adolescent whole grain consumers to the United States whole grain recommendation of 48g/d is presented in Table 3. Only 0.6% of children and 0.2% of the adolescents reached the target of three servings per day (48g/d), respectively. Approximately half of the children and adolescents consumed less than half a serving (8g/d) of whole grain per day (51.7% and 45.4%, respectively). Even when compared with the rough estimation of 43 g/d based on the Malaysian Dietary Guideline for Children and Adolescent outlined above, only 1.0% (n = 13) of children and 0.7% (n = 4) of adolescents met this recommended intake, respectively.

**Table 3:** Proportion of Malaysian children and adolescents whole grain consumer achieving different levels of the US whole grain intake recommendation.*

Servings Absolute intake	<1/2		≥1/2 to <1		≥1 to <2		≥2 to <3		≥3	
	<8 g/d		≥8 to <16 g/d		≥16 to <32 g/d		≥32 to <48 g/d		≥ 48 g/d	
	n	%	n	%	n	%	n	%	n	%
Children	665	51.7	453	35.2	138	10.7	22	1.7	8	0.6
Adolescent	252	45.4	233	42.0	58	10.5	11	2.0	1	0.2
Total	917	49.8	686	37.3	196	10.6	33	1.8	9	0.5

* U.S. Department of Agriculture (USDA) Dietary Guidelines for Americans (2010) [17]

## Discussion

To our knowledge, this is the first study to report on whole grain consumption patterns in Malaysian children and adolescents. Overall, whole grain consumption was extremely low with mean daily intakes less than 3g/d in both the children’s and adolescents samples. Furthermore, three quarters of the children and adolescents reported consuming no whole grain over the study period and among consumers, the majority consumed less than half a serving of whole grain per day (< 8g). RTEBCs were the main food contributor to whole grain intakes and wheat was the major grain source.

Similar to findings from other countries [9–10, 22, 26–28], whole grain intakes in Malaysian children and adolescents were considerably lower than quantitative whole grain recommendations (eg 48g/d in the US) and they also fell significantly short of the Malaysian recommendation (minimum 2 whole grain servings per day equating to an estimated 43g/d). However, the proportion of the Malaysian children and adolescents consuming whole grain was much lower than in the US and European populations (41–80%) [9–10, 26–28] and mean intakes were also considerably lower; mean whole grain intakes are approximately 9g/d in the US [26] while they range from about 4–54g/d in Europe [9–10, 22, 27, 28]. Whole grain intake data for children from other countries in the Southeast Asia region are lacking but intakes were much below the reported intakes in Singaporean adults in 2010 (26g/d) [23]. These cross country difference in intakes may be partially explained by methodological differences in study design and approaches used to identify and estimate the whole grain content of foods but it is likely there are other more significant contributor factors.

The demographic associations with whole grain intakes observed in the current study may give useful insights into potential barriers to the consumption of whole grain in Malaysia. For example, whole grain consumption was generally higher in the central region and in urban areas which may reflect greater availability of wholegrain food in these parts of Malaysia. It may also reflect the greater affluence concentrated in these areas and hence spending ability to buy wholegrain foods. Indeed, we observed a positive association between whole grain intake and monthly household income in both children and adolescents. Furthermore, there was a positive association between whole grain intake and education level (total sample of children only), which is likely to be linked with income. Similarly, in Ireland [9], and the UK [27], whole grain intake was positively associated with socio-economic status. The ethnic differences in whole grain intake whereby the highest intakes tended to be in Indian, Chinese and Bumiputera (native) children, may relate to the fact that a greater proportion of these ethnic groups were recruited in urban areas which in turn may reflect the greater availability to wholegrain foods and income as mentioned previously. Cultural background and dietary habits could be another influential factor [29]; however, the food sources of whole grain did not differ greatly between ethnicities with the exception of the Chinese where hot cereals made a greater contribution to whole grain intakes than in other ethnicities.

Overall, we observed only two predominant sources of whole grain in the diet of Malaysian children and adolescents; RTEBCs and hot cereals. RTEBCs were also a major contributor to whole grain intakes among children and adolescents in European (30–50%) [9–10, 27] and US (25%) populations [26], but in contrast to the current study, bread was a major source of whole grain in these two regions (20–60%) [9–10, 22, 26–28]. Notably, there was a minimal contribution from staple foods in the Malaysian diet including rice and noodles to whole grain intakes, a finding which differs from the Singaporean National Nutrition Survey [23], which reported that rice and bread are the major source of whole grain in the diets of Singaporean adults. The low consumption of wholegrain rice among Malaysian children could be related to its higher cost and lower availability, as well as taste acceptance compared to white rice. A previous study from Selangor demonstrated that consumers purchasing behavior for rice is affected by the flavor, taste and price [30]. Five kilograms of white rice (RM 12.99/ USD 3.42) is approximately twice the price of wholegrain rice (RM 30.00/ USD 7.89). In contrast, the apparent acceptance of wholegrain RTEBCs may be associated with greater palatability and convenience, despite higher costs. It has been reported previously that wholegrain RTEBCs are well accepted by a majority of the schoolchildren in Kuala Lumpur [31]. Further research is needed to understand consumer perceptions and behaviors in relation to wholegrain food choice.

Owing to their complete structure, whole grains provide a rich source of vitamins, minerals, fiber and antioxidants in the diet. Several cross sectional studies have demonstrated that children and adolescents consuming whole grain (even below levels outlined in quantitative dietary recommendations) have significantly higher intakes of dietary fiber, several B-vitamins and some minerals (mainly magnesium, iron, phosphorus and potassium) than non-consumers of whole grain [8–11]. Thus, increasing consumption of whole grain in Malaysian children and adolescents would appear to be an opportunity to improve fiber and micro-nutrient intakes in these age groups. Furthermore, studies in the US have shown a negative association between whole grain consumption and bodyweight in children and adolescents at intakes generally above 1 serving per day (>16g/d)[12–13, 32]. In the current study, no association between whole grain intake and body weight was observed (data not shown). This lack of association may be due to the overall low whole grain intake in the majority of the sample as only 13% achieved a daily intake above 16g/d.

Considering the minimal whole grain consumption levels in Malaysia and the epidemiological evidence showing a positive effect of whole grain consumption on nutritional health and bodyweight in other populations, it is imperative to find ways to promote whole grain consumption among children and adolescents. As food preferences are established in childhood and often track into adulthood, it is a critical age at which to educate children about whole grains and to expose them to a range of whole grain foods. A recent study of 384 children (10–11 years) from Kuala Lumpur reported that 70% of the children had a low knowledge level on whole grains while the majority had a neutral attitude towards whole grain [33]. Encouragingly however, knowledge of whole grains was positively correlated with attitude and with practice, suggesting a potential role for education-based strategies in improving children’s attitudes towards whole grains and eventual consumption levels. Nevertheless, nutrition modules are currently not a compulsory part of the standard teaching curriculum in Malaysia schools. While the Nutrition Society of Malaysia tries to promote healthy eating including whole grain among children in selected schools (eg through the Health Kids Programme) [34], there is a need for more concerted efforts in schools across all parts of the country. In both the US [35] and Singapore [36], as part of their School Meals Programme, school canteens are strongly encouraged to offer whole grain alternatives to children. There are currently no similar school breakfast or school lunch programmes in place in Malaysia. Nonetheless, education on whole grain consumption can still be carried out through the school canteens. In Singapore, the health promotion board has extended its efforts to promote whole grain consumption beyond school-based programmes to out of home eating locations through its Healthier Hawker Centre Programme [37]. This programme aims to increase the availability of whole grain noodles and rice in traditional dishes offered by hawker centres without increasing the cost or compromising taste. As hawker centres are a common place of food consumption in children and especially adolescents in Malaysia, consideration could be given to a similar programme in Malaysia.

In Malaysia and elsewhere, a legally endorsed definition of both whole grain and wholegrain food is lacking. Recently, a standardized definition of a wholegrain food was proposed by an expert panel of US and European nutrition scientists, consumer educators and legislators suggesting that a wholegrain food should contain a minimum 8g whole grain per 30g serving [38]. Steps should be taken by the regulatory authority in Malaysia to approve a definition of whole grain and a wholegrain food, as well as mandating that foods labeled as whole grain should declare the amount of whole grain in the product. Such developments may encourage manufacturers to make more wholegrain foods available to consumers and to reformulate existing foods to increase the whole grain content. Furthermore, the development of a distinctive food label logo which signifies that a food is a good source of whole grain, as currently used in the successful Danish whole grain campaign [22] may aid consumers in identifying and selecting wholegrain foods.

This present study has some strengths and weaknesses that should be acknowledged. To our knowledge, this is the first study to assess the consumption of whole grains among Malaysian children and adolescents in a large national sample covering a wide age range from 6 to 17 years old, from both rural and urban areas, as well as different ethnic groups. In addition, all of the whole grain foods were recorded at the brand level. However, the use of a 2-day recall/record may not reflect habitual whole grain consumption in all participants and as with all dietary surveys there is a possibility of under-reporting or over-reporting of food intake, thus whole grain intakes. Furthermore, parents of the younger children may not have accurately recalled all foods consumed by their children especially foods consumed outside the home. In addition, it is possible that some whole grain foods were not included in the analysis if the food label did not clearly indicate that the product contained whole grain. Lastly, the present study was cross-sectional, thus statements about the causality of associations are not possible.

In conclusion, the study presents important information on whole grain consumption in Malaysian children and adolescents, which can serve to provide more targeted interventions to increase whole grain consumption. A minority of Malaysian children and adolescents are currently consuming whole grain and intakes among consumers fall significantly short of recommendations. Collaborative efforts are needed by all stakeholders including policy makers, healthcare providers, educators and the food industry to increase whole grain consumption among Malaysian children and adolescents. In addition, more studies, preferably of a longitudinal and interventional nature, are needed to provide more evidence for associations between whole grain consumption and health outcomes among children and adolescents.
