# Correction: Whole Grain Intakes in the Diets Of Malaysian Children and Adolescents – Findings from the MyBreakfast Study

**DOI:** 10.1371/journal.pone.0142763

**Published:** 2015-11-06

**Authors:** Norimah AK, H. C. Koo, Hamid Jan JM, Mohd Nasir MT, S. Y. Tan, Mahenderan Appukutty, Nurliyana AR, Frank Thielecke, Sinead Hopkins, M. K. Ong, C. Ning, E. S. Tee

The sixth author’s name is spelled incorrectly. The correct name is: Mahenderan Appukutty. The correct citation is: AK N, Koo HC, JM HJ, MT MN, Tan SY, Appukutty M, et al. (2015) Whole Grain Intakes in the Diets Of Malaysian Children and Adolescents–Findings from the MyBreakfast Study. PLoS ONE 10(10): e0138247. doi:10.1371/journal.pone.0138247

There are errors in the Funding section. The correct funding information is as follows: The Nutrition Society of Malaysia received an unrestricted research grant from Cereal Partners Worldwide, Switzerland and Nestleé R&D Center, Singapore. This financial support was provided in the form of salaries for research assistants but the funders did not have any additional role in the study design, data collection and analysis or decision to publish. The specific roles of these authors are articulated in the “author contributions” section.
